# Effect of Hochuekkito (Buzhongyiqitang) on Nasal Cavity Colonization of Methicillin-Resistant *Staphylococcus aureus* in Murine Model

**DOI:** 10.3390/medicines5030083

**Published:** 2018-08-01

**Authors:** Masaaki Minami, Toru Konishi, Toshiaki Makino

**Affiliations:** 1 Department of Bacteriology, Graduate School of Medical Sciences, Nagoya City University, 1 Kawasumi, Mizuho-ku, Nagoya 467-8601, Japan; 2 Department of Pharmacognosy, Graduate School of Pharmaceutical Sciences, Nagoya City University, 3-1 Tanabe-Dori, Mizuho-ku, Nagoya 467-8603, Japan; c142902@ed.nagoya-cu.ac.jp (T.K.); makino@phar.nagoya-cu.ac.jp (T.M.)

**Keywords:** MRSA, Hochuekkito, Japanese traditional Kampo medicine, murine colonization model

## Abstract

**Background:** Methicillin-resistant *Staphylococcus aureus* (MRSA) infections are largely preceded by colonization with MRSA. Hochuekkito is the formula composing 10 herbal medicines in traditional Kampo medicine to treat infirmity and to stimulate immune functions. We evaluated the efficacy of hochuekkito extract (HET) against MRSA colonization using a nasal infection murine model. **Methods:** We evaluated the effects of HET as follows: (1) the growth inhibition by measuring turbidity of bacterial culture in vitro, (2) the nasal colonization of MRSA by measuring bacterial counts, and (3) the splenocyte proliferation in mice orally treated with HET by the ^3^H-thymidine uptake assay. **Results:** HET significant inhibited the growth of MRSA. The colony forming unit (CFU) in the nasal fluid of HET-treated mice was significantly lower than that of HET-untreated mice. When each single crude drug—Astragali radix, Bupleuri radix, Zingiberis rhizoma, and Cimicifugae rhizome—was removed from hochuekkito formula, the effect of the formula significantly weakened. The uptake of ^3^H-thymidine into murine splenocytes treated with HET was significantly higher than that from untreated mice. The effects of the modified formula described above were also significantly weaker than those of the original formula. **Conclusions:** Hochuekkito is effective for the treatment of MRSA nasal colonization in the murine model. We suggest HET as the therapeutic candidate for effective therapy on nasal cavity colonization of MRSA in humans.

## 1. Introduction

*Staphylococcus aureus* infection, such as surgical site infection, is a common hospital-associated infectious disease. It causes the extension of hospital stays and increases the costs of health-care [1]. The increasing rates of clinical isolates of *S. aureus* worldwide are methicillin-resistant [2]. The attributable mortality of *S. aureus* septicemia infection is about 20% for methicillin-sensitive strains and about 30% for methicillin-resistant *S. aureus* (MRSA) [3]. The development of new effective medication is desired for the improvement of morbidity and mortality regarding *S. aureus* infection.

Nasal colonization is an important risk factor for *S. aureus* infection. It is associated with up to 13-fold increased risk of *S. aureus* infection [4]. A study of nosocomial *S. aureus* bacteremia demonstrated nasal colonization on admission in most cases [5]. Nasal colonization is the predecessor to infection because the infecting strain was identical to the isolated colonizing strain before infection in four-fifths of *S. aureus* septicemia cases [6]. Decolonization therapy reduces the risk of healthcare-associated *S. aureus* infection in high-risk settings such as surgery, supporting the hypothesis that colonization leads to infection [7].

Traditional Chinese medicine (TCM) is one of the most popular alternative, complementary therapies worldwide [8]. In Japan, Kampo medicine, which is the traditional medicine developed from ancient Chinese medicine, is recognized as an effective alternative medicine against several diseases [9,10]. Hochuekkito (Buzhongyiqitang) is a formula in both traditional Japanese Kampo medicine and Chinese medicine. This formula comprises 10 crude drugs shown in Table 1. Hochuekkito extract (HET) has been used to treat severe infirmity such as weakness and loss of appetite of the elderly [11]. As HET is a popular alternative medicine in Japan, limited scientific evidence is available on the use of HET for the treatment of MRSA colonization [12,13]. Thus, the clarification of the precise mechanism of Hochuekkito efficacy against MRSA colonization has been desired.

In the present study, we evaluated the efficacy of HET against MRSA colonization using a nasal infection murine model. Furthermore, we also evaluated the efficacy of the constitutive crude drug of HET against MRSA and immunological activity of murine splenocytes from HET-treated mice.

## 2. Materials and Methods

### 2.1. Bacterial Strains and Culture Condition

MRSA (ATCC_BAA-1556 (FPR3757)) (American Type Culture Collection, Rockville, MD, USA) was used in this study. After overnight pre-incubation on TSAII sheep blood agar (Nihon Becton Dickinson, Tokyo, Japan), a fresh colony of bacteria was cultured for 16 h at 37 °C. The bacteria were harvested by centrifugation and re-suspended in sterile Luria–Bertani (LB) medium (Becton Dickinson, Franklin Lakes, NJ, USA). Bacterial density was determined by measuring the absorbance at 600 nm (A600). The bacterial suspension was then diluted with LB to 10^6^ CFU (colony forming unit)/mL using a standard growth curve to relate measured A600 to bacterial concentration. The bacteria were cultured at 37 °C and A600 was measured at every 2 h.

### 2.2. Crude Drugs and Exteact Preparation

**Figure 1:**
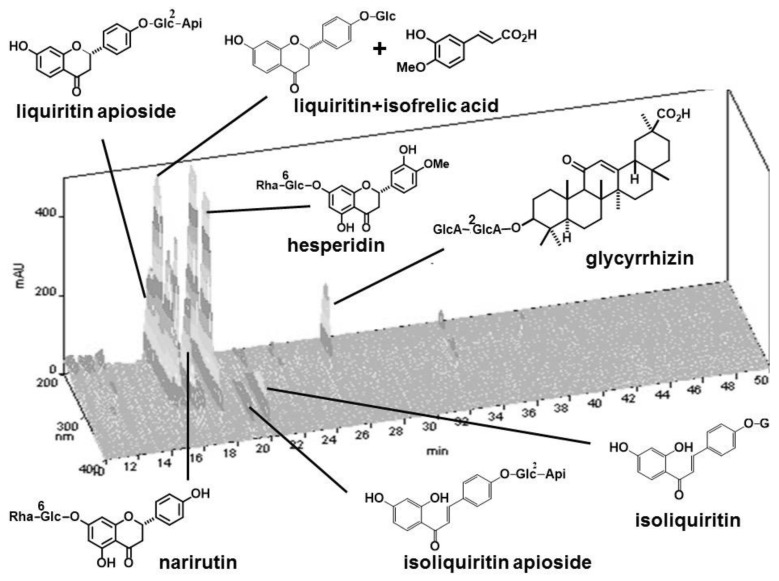
HPLC fingerprint of hochuekkito extract (HET). Compounds were identified by comparison of the retention times of the UV spectra with those of standard compounds.

Astragali radix (lot number, 6C30M), 4.0 g of Ginseng radix (5D25), 4.0 g of Atractylodes rhizome (3J07M), 3.0 g of Angelicae radix (5G06M), 2.0 g of Zizyphi fructus (5G07M), 2.0 g of Aurantii nobilis pericarpium (6B16M), 2.0 g of Bupleuri radix (6C15M), 1.5 g of Glycyrrhizae radix (6B22), 1.0 g of Cimicifugae rhizome (0F28M), and 0.5 g of Zingiberis rhizome (5G07M). These cut crude drugs were purchased from Daiko Shoyaku (Nagoya, Japan) and standardized by Japanese Pharmacopoeia 17th Edition [14]. Voucher specimens of each single crude drug were deposited in the Department of Pharmacognosy, Graduate School of Pharmaceutical Sciences, Nagoya City University. The mixture of the above crude drugs was boiled in 20-times weight of water for 30 min, and filtered. The decoction was lyophilized to yield powdered extract (HET, the ratio of the extract yielded was 36%). A fingerprint pattern of this HET was created as follows. HET (50 mg) was suspended with MeOH (1 mL) and sonicated for 30 min. The supernatant (30 μL) was injected to HPLC with the following conditions: system, Shimadzu LC-10A*_VP_* (Kyoto, Japan); column, TSK-GEL ODS-80_TS_ (4.6 × 250 mm, Tosoh, Tokyo, Japan); mobile phase, 0.05 M AcOH–AcONH_4_ buffer (pH 3.6)/CH_3_CN 90:10 (0 min)–45:55 (40 min), linear gradient; flow rate, 1.0 mL/min; column temperature, 40 °C; and detection, 200–400 nm by a photodiode array detector. Some peaks were identified by the retention times and UV spectra of the standard compounds. The fingerprint chromatogram of HET extract is shown in Figure 1. HET was suspended in distilled water to prepare the stock solution at a concentration of 0.1 g/mL, and kept at −20 °C until use. From the 10 crude drugs of hochuekkito formula, each single crude drug was removed to make 10 kinds of modified hochuekkito formula containing 9 crude drugs. The extracts of the modified formula were prepared in the same way.

### 2.3. Murine Model of Bacterial Nasal Infection

**Figure 2:**
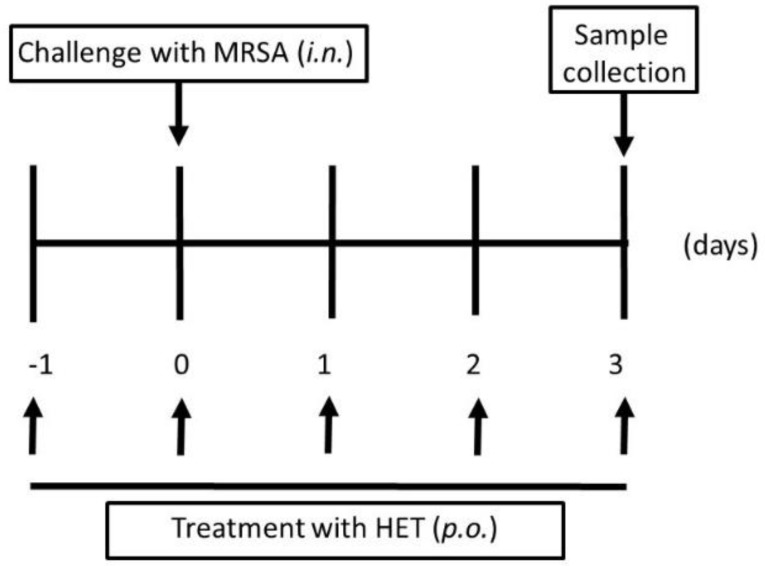
Protocols for murine experiments of methicillin-resistant *Staphylococcus aureus* (MRSA) colonized model. In the infected group, 1 × 10^7^ colony forming unit (CFU) bacteria were injected into both nostrils of mice using a 29 gauge needle at day 0. In the hochuekkito extract (HET)-treated group, mice were administrated with HET *p.o.*

This study was approved by the Animal Experiment Committee of Graduated School of Medical Sciences of Nagoya City University in accordance with the guidelines of the Japanese Council on Animal Care. Ethical approval code: H28M-05. Date of approval: 2 March 2016. Mice were purchased from Japan SLC (Hamamatsu, Japan). The ability of the colonized effect of MRSA in mice after nasal inoculation was assessed using a previous procedure [15]. In brief, bacteria were harvested after 16 h of growth on TSAII sheep blood agar, and were mixed in 1 mL of phosphate buffered saline (PBS, pH 7.2, 0.15 M), then centrifuged at 2000× *g* for 2 min. The pellets were diluted in 100 µL PBS to 1 × 10^7^ CFU, and then inoculated into both nostrils of inbred six-week-old female Balb/c mice using a micropipette. The number of CFU inoculated was verified for each experiment by plating the bacteria on TSAII sheep blood agar and counting CFU. Mice were observed daily. In the HET-treated group, mice were administered with HET (0.85, 1.7, or 3.4 g/kg body weight/day body weight, which were equivalent to 5, 10, and 20 times the dosage of humans, respectively) on days −1, 0, 1, 2, and 3 after the bacterial inoculation (Figure 2). The mice in the control group were given PBS without infection.

### 2.4. Nasal Lavage Cultures

The procedure of nasal cultures was described elsewhere [16]. In brief, the mice were sacrificed by CO_2_ inhalation. After that, the external noses, oral cavity, and head were disinfected with a moist alcohol swab and allowed to dry. Nasal lavage was performed with 200 µL of PBS. The recovered fluid was then serially diluted, and 10 μL of each dilution was plated onto TSAII sheep blood agar plates. The plates were incubated for 24 h, and then colonies of bacteria were counted. The results were quantified as the number of CFU/mL.

### 2.5. Determination of Splenocyte Proliferative Response

The oral administration protocol for this assay was done in almost the same manner for bacterial nasal infection, except for no-infection with MRSA. After the mice were sacrificed by CO_2_ inhalation, the spleen was removed aseptically, and splenocytes were filtered and cultured in RPMI 1640 (Wako Pure Chemical Industry, Osaka, Japan) containing 5% fetal calf serum (FBS, Sigma-Aldrich, St. Louis, MO, USA). At 20 h prior to the culmination of the splenocyte culture, ^3^H-thymidine (2.0 Ci/mmol; PerkinElmer, Waltham, MA, USA) was added into the medium, and the cells were further incubated for 4 h. Then, the cells were adsorbed on 0.45-μm membrane filters, washed with distilled water, and then dried. The filters were transferred to vials filled with liquid scintillator cocktail (Ultima Gold, Perkin Elmer, Inc., Waltham, MA, USA), and the radioactivity was measured by using a liquid scintillation counter (LSC-6100, Hitachi Aloka Medical, Tokyo, Japan). The results are given as disintegrations per minute (DPM).

### 2.6. Statistical Analysis

All statistical analyses were conducted using Tukey/Bonferroni’s multiple comparison test for differences among multiple groups (EZR version 1.36, http://www.jichi.ac.jp/saitama-sct/SaitamaHP.files/statmedEN.html). Values less than 0.01 indicated statistical significance.

## 3. Results

### 3.1. Bacterial Growth Inhibitory Effect

**Figure 3:**
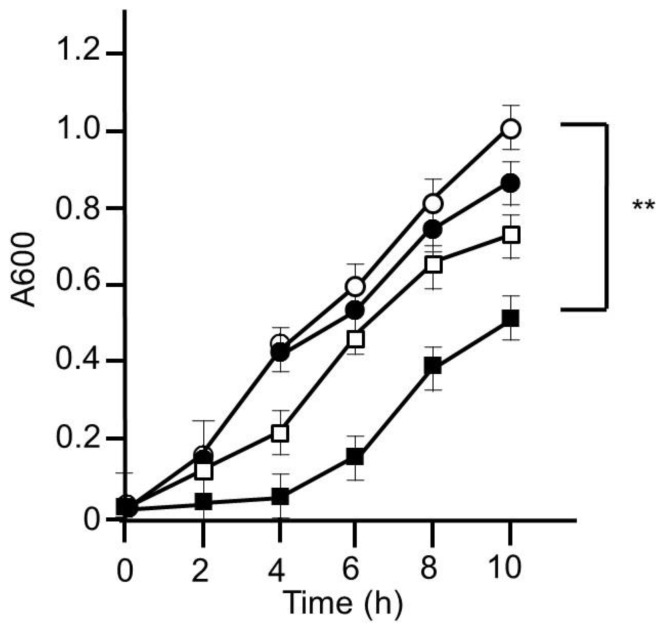
Bacterial growth inhibitory effect of hochuekkito extract (HET). MRSA was cultured on Luria–Bertani (LB) medium with or without HET for 10 h. The bacterial growth was evaluated by measuring absorbance at 600 nm. Open circle, closed circle, open square, and closed square exhibited HET 0, 0.1, 1, and 10 mg/mL, respectively. Data shown represent the mean ± S.D. (*n* = 6). ** *p* < 0.01 by Tukey/Bonferroni’s multiple comparison test.

First of all, we tried to evaluate whether or not HET could inhibit the growth of MRSA. MRSA was grown in LB medium with or without HET, and the inhibitory ability of bacterial growth was assessed. As expected, HET (10 mg/mL) significantly inhibited the growth of MRSA (*p* < 0.01). We confirmed that this inhibitory ability was in dose- and time-dependent manners (Figure 3).

### 3.2. Murine Nasal Infection Model

**Figure 4:**
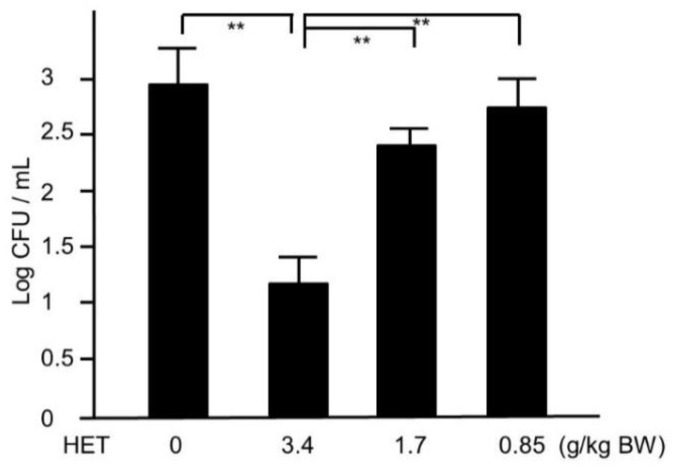
The colonies of MRSA in hochuekkito extract (HET)-treated and untreated murine nasal lavage. The nasal fluids were inoculated on TSAII sheep blood agar and incubated for 24 h. Comparisons of colony count between HET-treated and untreated mice were performed. Data represent the mean ± S.D. (*n* = 6). ** *p* < 0.01 by Tukey/Bonferroni’s multiple comparison test.

**Figure 5:**
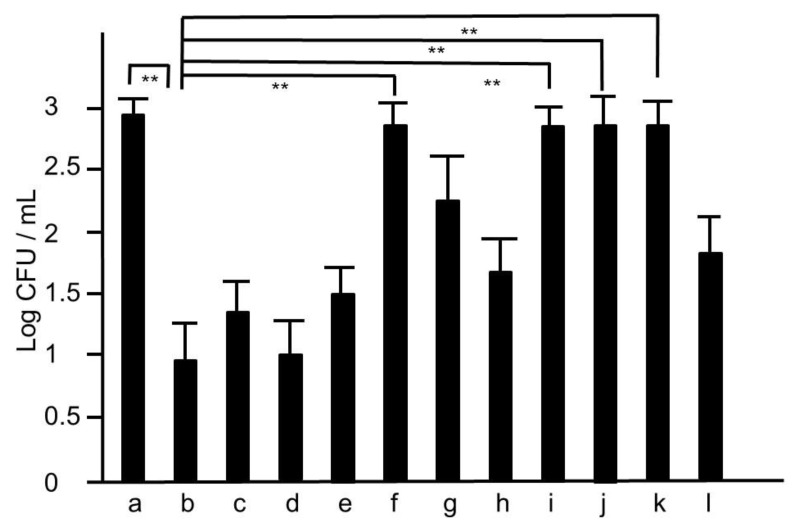
The colonies of MRSA of nasal lavage collected from mice treated with the extracts of modified hochuekkito formulas. The nasal fluids were inoculated on TSAII sheep blood agar and incubated for 24 h. a: untreated, b: hochuekkito extract (HET)-treated, c: aurantii nobilis pericarpium-removed HET, d: zizyphi fructus-removed HET, e: angelicae radix-removed HET, f: zingiberis rhizome-removed HET, g: Atractylodes rhizome-removed HET, h: Ginseng radix-removed HET, i: astragali radix-removed HET, j: bupleuri radix-removed HET, k: cimicifugae rhizome-removed HET, l: glycyrrhizae radix-removed HET, respectively. Dosage of HET was 3.4 g/kg/day, and those of the extracts of other modified hochueekito formulas were equivalent to this dosage. Data represent the mean ± S.D. (*n* = 6). ** *p* < 0.01 by Tukey/Bonferroni’s multiple comparison test.

Next, we tried to assess whether HET would provide in vivo effects against MRSA. Four days after nostril infection of MRSA, we evaluated the bacterial colony counts in murine nose. The CFUs of HET-treated murine nasal lavage were lower than those of HET-untreated mice in dose-dependent manners, and the group treated with 3.4 g/kg/day exhibited statistical significance (*p* < 0.01) (Figure 4). In order to find the active components in the hochuekkito formula, we prepared the extracts of the modified formulas, which contain nine crude drugs. The extracts of modified hochuekkito formulas—that is, Astragali radix, Bupleuri radix, Zingiberis rhizoma, or Cimicifugae rhizome—exhibited significantly lower activities than HET, respectively (*p* < 0.01) (Figure 5).

### 3.3. Splenocyte Proliferative Activity in HET-Treated Mice

**Figure 6:**
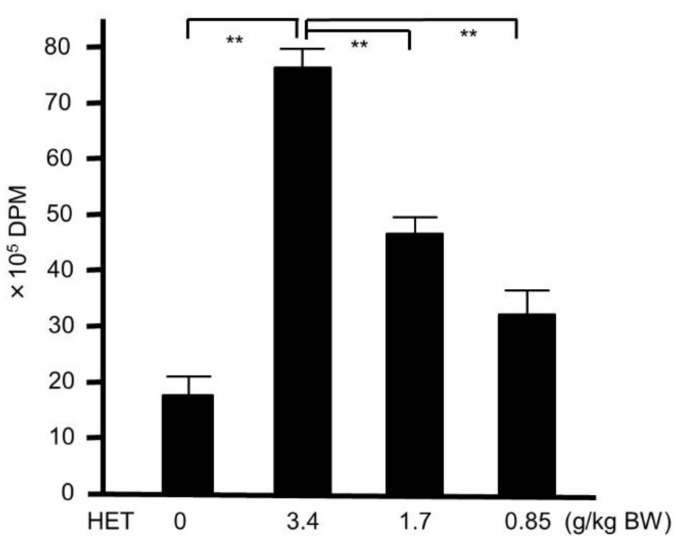
^3^H-thymidine-uptake assay in hochuekkito extract (HET)-treated and untreated murine splenocyte. Six-week-old female Balb/c mice were administrated with HET for four days, and the splenocyte were collected. Data represent the mean ± S.D. (*n* = 6). ** *p* < 0.01 by Tukey’s/Bonferroni multiple comparison test. DPM—disintegrations per minute.

**Figure 7:**
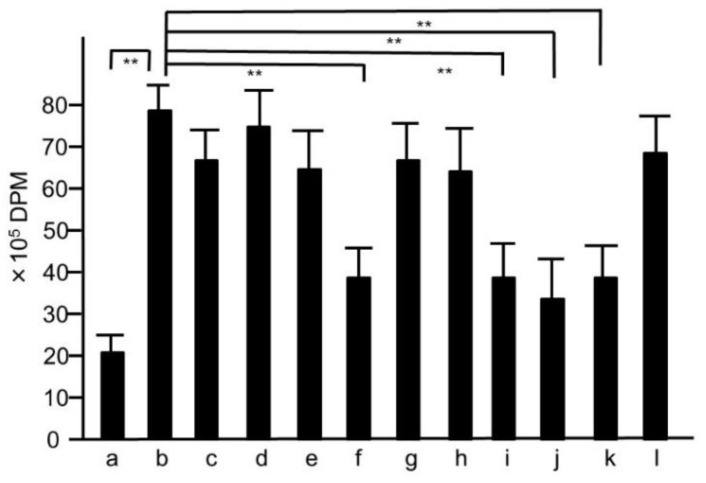
^3^H-thymidine-uptake assay of the splenocytes collected from mice treated with the extracts of modified hochuekkito formulas. Six-week-old female Balb/c mice were administrated with hochuekkito extract (HET) for four days, and splenocytes were collected. Symbols of a–l and the dosages of the samples were as same as those shown in Figure 5. Data represent the mean ± S.D. (*n* = 6). ** *p* < 0.01 by Tukey/Bonferroni’s multiple comparison test.

We also studied the activity of splenocyte in mice treated with HET, because splenocytes play major roles in murine bacterial infection models. To determine whether or not the activity of the splenocytes collected from HET-treated mice was elevated, we performed ^3^H-thymidine uptake analysis. As shown in Figure 6, the uptake of ^3^H-thymidine into splenocytes collected from mice orally treated with HET was significantly (*p* < 0.01) higher than that from untreated mice in dose-dependent manners. The extracts of modified hochuekkito formulas—that is, Astragali radix, Bupleuri radix, Zingiberis rhizoma, or Cimicifugae rhizome—exhibited significantly lower ^3^H-thymidine uptake compared with HET (*p* < 0.01) (Figure 7).

## 4. Discussion

In this study, we tried to clarify that HET would be effective for the eradication in the MRSA-colonized murine model. Our results showed that the turbidity of the bacteria increases over time at the start and that the turbidity decreases compared with the control when HET is added, so this is the growth suppressing effect. After MRSA nasal infection, HET-treated mice showed a reduction of MRSA colonization in murine nose and the upregulation of murine splenocyte activity. Furthermore, we demonstrated that four crude drug components of HET—Astragali radix, Bupleuri radix, Zingiberis rhizoma, and Cimicifugae rhizome—affected the eradication of MRSA. Our results suggest that HET can play a crucial part in protection against MRSA colonization in the mouse model.

Several studies of HET on microbial infections have been investigated. In a small-scale clinical trial about MRSA infection, eradication of MRSA was successful when HET was administered to five MRSA carriers’ patients [12]. Another study showed that when HET was administered to 34 asymptomatic patients from which MRSA was isolated from urine, MRSA was not isolated from urine in 12 patients, and 10 patients decreased the bacterial volume to less than 1/100 [13]. Other human clinical trials in lung *Mycobacterium avium* complex patients with HET for six months resulted in weight gain and increased serum albumin value without a tendency for infectious disease to exacerbate on chest radiograph [17]. Even in healthy elderly humans, natural killer (NK) cell activity increased at 30 days and 120 days after administration of HET. The serum interferone (IFN)-γ activity also increased [18]. A clinical large-scale trial to confirm the effect of HET on MRSA carriage of human nasal cavity is desired from the investigation of the effect of HET on these bacterial colonizations and chronic infections for humans and our experimental results.

Non-human experimental studies also revealed the efficacy of HET against bacterial infection. When *Listeria monocytogenes* was infected intraperitoneally in mice, HET showed an increase in polynuclear leukocytes and macrophages in the spleen. HET also confirmed renewal of phagocytic capacity of *L. monocytogenes* in intraperitoneal macrophages [19]. In a mouse infected with *L. monocytogenes*, HET showed a decrease in bacterial quantities in Peyer’s patches, lymph nodes, and liver. HET showed increased phagocytosis of liver macrophages against bacteria. It also showed an increase in IFN-γ producing cells in intraepithelial lymphocytes [20]. When HET was administrated in *L. monocytogenes* infected infant mice, the amount of *L. monocytogenes* in the liver and spleen decreased. Activation of IFN-γ producing CD4 T cells enhanced IFN-γ activity. The ability of macrophages to present antigen by MHC class II expression is enhanced [21]. In vitro experiments inhibited the growth of *Helicobacter pylori* at a concentration of HET 2.5 mg/mL. In addition, the amounts of bacteria in the stomach were decreased in mice by oral administration of HET in an in vivo experiment. Furthermore, the expression of IFN-γ in the gastric mucosa was elevated [22]. As our bacterial growth study showed that HET suppressed the MRSA in a dose-dependent manner, HET may have a bacterial inhibitory effect regardless of bacterial species. By infecting mice with *Brucella abortus*, causing a chronic fatigue syndrome, the combined effect of HET and IFN-γ increased the activity of thymic NK cells [23]. HET treatment increased the expression of human monocyte-like THP-1 cells on the cell surface of toll-like receptor (TLR) 4, resulting in an increase in receptors responsive to gram-negative bacteria. From this result, it is also considered to activate the protective effect against pathogenic bacteria [24].

Several reports about viral infection also showed that HET was effective for respiratory viral infection via immunomodulation system such as cytokines. As the nasal cavity belongs to respiratory organs, the anti-infective effect against respiratory infections may give some hint to the eradication of nasal colonization. HET administration resulted in improvement of survival rate and survival time with the mouse influenza virus infection. We also found suppression of viral load in bronchoalveolar lavage fluid (BALF) [25]. An increase in lung interleukin (IL)-1β and tumor necrosis factor-α was observed in combination with HET and osetamivir for influenza A virus-infected mice. In addition, hyperactivity of mouse alveolar macrophages was also observed [26]. In the mouse influenza virus infection model, virus titres decreased in BALF with HET administration. HET also stimulated not only the release of type 1 IFN in the lung, but also the anti-inflammatory response derived from granulocyte macrophage colony-stimulating factor. Furthermore, the defensin expression of the antimicrobial peptide was also increased [27]. When mice were infected with rhinovirus, it was thought that HET inhibited intracellular migration of rhinovirus by decreased expression of intercellular adhesion molecule-1 of airway epithelial cells. It also inhibited IL-1β, IL-6, and IL-8 secretion from respiratory epithelial cells. In our results, glycyrrhizae radix that contains glycyrrhizin was not prominent in the effect of HET, but it is reported that glycyrrhizin reduced viral antibody titter [28].

Splenocytes are the major immunomodulation system against bacterial infection [29]. Several crude drug are known to promote immunostimulation of spleen cells. Atractylodes rhizome extract promotes T cell activity by expressing CD28 of T cells in spleen [30]. It also promotes secretion of IL-2, 6, 10, and T cell differentiation via phosphorylation of extracellular signal-regulated kinases [31]. In addition, it reduces the IFN-γ secretion from T cells in helper T (Th) 1 cells and promotes the IL-4 secretion in Th2 cells [32]. Zingiberis rhizoma extract stimulates CD8+ T cells of splenocytes [33,34]. In addition, it is involved in the TLR2/NF-κB pathway by suppressing the expression of TLR2/NF-κB p65 in lung tissue with mouse pneumococcal infection [35]. Bupleuri radix extract reduces the Th1 subunit and increases the Th2 subunit of peripheral blood [36]. It also has B cell mitogenic activity in spleen cells [37]. Moreover, it also has antimicrobial and antiviral action [38]. Our findings also suggested that these constitutional crude drugs of HET were involved in the activation of spleen cells.

Furthermore, three kinds of active crude drugs, Bupleuri radix, Cimicifugae rhizoma, and Zingiberis rhizome, in hochuekkito belong to superfices-syndrome relieving drugs in Kampo medicinal theory, which means that it excludes the *evils* of the inner surface of the body. This traditional medicinal theory may explain that these three crude drugs have the effect of nasal infection of bacteria.

## 5. Conclusions

In summary, HET is significantly effective for the treatment of nasal cavity colonization of MRSA in the murine model. We suggest HET as the therapeutic candidate for effective therapy on nasal cavity colonization of MRSA in humans.

## Figures and Tables

**Table 1:** Composition of hochuekkito.

Name of Crude Drug	Origin	Daily Dose (g)
Astragali radix	The dried root of *Astragalus propinquus* Schischkin, Fabaceae	4.0
Ginseng radix	The dried root of *Panax ginseng* C.A. Mayer, Araliaceae	4.0
Atractylodes rhizome	The dried rhizome of *Atractylodes japonica* Koidzumi ex Kitamura, Asteraceae	4.0
Angelicae radix	The dried root of *Angelica acutiloba* (Siebold & Zucc.) Kitag., Apiaceae	4.0
Zizyphi fructus	The dried fruit of *Ziziphus jujuba* Miller, Rhamnaceae	3.0
Aurantii nobilis pericarpium	The dried ripe fruit skin of *Citrus reticulata* Blanco, Rutaceae	2.0
Bupleuri radix	The dried root of *Bupleurum falcatum* Linné, Apiaceae	2.0
Glycyrrhizae radix	The dried root and stolon of *Glycyrrhiza uralensis* Fisher, Fabaceae	1.5
Cimicifugae rhizome	The dried rhizome of *Actaea simplex* (DC.) Wormsk. ex Prantl, Ranunculaceae	1.0
Zingiberis rhizome	The dried rhizome of *Zingiber officinale* Roscoe, Zingiberaceae	0.5

Daily doses of crude drugs in hochuekkito are in Japanese Pharmacopoeia 17th Edition [14].
